# The Role of D-3-Phosphoglycerate Dehydrogenase (PHGDH) in Chemoresistance in Ovarian Cancer: Molecular Insights and Therapeutic Potential

**DOI:** 10.3390/ijms27219535

**Published:** 2026-10-25

**Authors:** Jesús Omar Velázquez-Moreno, María de la Luz Mejía-Aguayo, Felix De Dios Padre Casarrubias-Jaimes, Marcela Angélica De la Fuente-Hernández, Daniel de Jesús Apolonio-Sánchez, Rodrigo Sosa-León, Rosa María Álvarez-Gómez, Marco Antonio Leyva-Vázquez, Abraham Pedroza-Torres, Olga Lilia Garibay-Cerdenares

**Affiliations:** 1 Laboratorio de Biomedicina Molecular, Facultad de Ciencias Químico-Biológicas, Universidad Autónoma de Guerrero, Av. Lázaro Cárdenas S/N Sede Sur, El Centenario, Chilpancingo 39090, Mexico; omar.velazquezm2703@gmail.com (J.O.V.-M.); felixito26@icloud.com (F.D.D.P.C.-J.); dapolonnio@gmail.com (D.d.J.A.-S.); leyvamarco13@gmail.com (M.A.L.-V.); 2 Clínica de Cáncer Hereditario— Instituto Nacional de Cancerología, Av. San Fernando 22, Belisario Domínguez Secc 16, Tlalpan, Ciudad de Mexico 14080, Mexico; merry.luz1894@gmail.com (M.d.l.L.M.-A.); marcela.dlfh@gmail.com (M.A.D.l.F.-H.); sosaleon.roy@gmail.com (R.S.-L.); ralvarezg@incan.edu.mx (R.M.Á.-G.); 3 Programa Investigadores por México—Secretaría de Ciencia, Humanidades, Tecnología e Innovación (SECIHTI), Av. Insurgentes Centro 1582, Crédito Constructor, Benito Juárez, Ciudad de Mexico 03940, Mexico

**Keywords:** ovarian cancer, PHGDH, metabolism, platinum-resistance

## Abstract

Ovarian cancer (OC) is often diagnosed at advanced stages, and resistance to platinum-based chemotherapy remains one of the leading causes of treatment failure. This resistance is sustained in part by metabolic plasticity, particularly through the de novo serine synthesis pathway (SSP), orchestrated by D-3-phosphoglycerate dehydrogenase (PHGDH), which diverts glycolytic carbon toward serine, glycine, and one-carbon (1C) units that fuel nucleotide biosynthesis, epigenetic regulation, and redox homeostasis. Elevated PHGDH expression in OC is associated with advanced International Federation of Gynecology and Obstetrics (FIGO) stage and poorer overall survival; conversely, a subset of tumors downregulates PHGDH at relapse and shifts toward NAD^+^-dependent pathways that sustain poly(ADP-ribose) polymerase (PARP) activity and DNA repair, creating a distinct therapeutic vulnerability. Rather than a contradiction, this bidirectional behavior reflects the metabolic plasticity through which OC adapts to treatment pressure. Therefore, this review analyzes the context-dependent role of PHGDH in OC, the mechanistic basis of divergent associations with therapy resistance, and the current status of pharmacological inhibitors. Preclinical evidence indicates that PHGDH inhibition with CBR-5884 increases oxidative stress, reduces stemness, and restores platinum sensitivity in PHGDH-high models. We further identify the relationship between PHGDH and radiotherapy response as an unexplored question in OC, despite evidence of SSP-dependent radioresistance in other tumor types. Together, these observations position PHGDH as an actionable, but biomarker-dependent, metabolic target in OC.

## 1. Introduction

Ovarian cancer (OC) is a heterogeneous and lethal gynecologic malignancy [1,2,3] that is frequently diagnosed at advanced stages and associated with poor prognosis [4]. The standard treatment for OC is based on cytoreductive surgery followed by platinum-based chemotherapy, usually in combination with paclitaxel [5]. Although this regimen achieves high initial response rates, most patients relapse due to the development of chemoresistance [6,7]. Of note, platinum resistance constitutes a therapeutic barrier and reduces survival to less than 15 months after its development [5,6,7]. These clinical limitations highlight the need to understand the molecular mechanisms responsible for treatment failure.

Metabolic reprogramming has been recognized as a hallmark of cancer that contributes to tumor survival in the face of chemotherapy [8,9]. Tumor cells modify central carbon metabolism to produce biomass and maintain redox balance, thereby ensuring their survival [10,11]. In this context, serine biosynthesis, which fuels one-carbon (1C) metabolism, enables nucleotide synthesis, the antioxidant response, and epigenetic regulation [12,13].

D-3-phosphoglycerate dehydrogenase (PHGDH), an enzyme that limits serine synthesis, has gained prominence as a therapeutic target for regulating tumor progression and drug resistance [14,15,16,17,18]. Its overexpression has been documented in various cancers [19,20,21,22] and has been linked to increased antioxidant capacity, increased production of reduced nicotinamide adenine dinucleotide phosphate (NADPH) and glutathione (GSH), and resistance to oxidative stress [14,23,24].

In OC, PHGDH expression has been linked to platinum resistance and a poorer clinical prognosis [14,25]. However, recent evidence suggests that PHGDH regulation is dynamic and context-dependent. In certain platinum-resistant tumors, decreased PHGDH following relapse is associated with metabolic reprogramming toward NAD^+^-dependent pathways, which supply the substrate PARP requires for poly(ADP-ribosyl)ation, sustaining DNA repair and tumor survival [6]. This adaptive downregulation contrasts with the effect of therapeutically inhibiting PHGDH in tumors where it remains overexpressed. Preclinical studies have demonstrated that pharmacological inhibition or genetic silencing of PHGDH can increase platinum sensitivity and enhance oxidative stress in cellular models [26,27].

Although PHGDH and the serine synthesis pathway (SSP) have been reviewed extensively in the general context of cancer metabolism, their specific contribution to chemoresistance in OC has not been examined in an integrated manner. The present review addresses this gap by focusing on OC and, in particular, on the apparent paradox that has emerged from recent evidence: whereas PHGDH overexpression is associated with platinum resistance, poor prognosis, and enhanced antioxidant and 1C capacity in a substantial subset of tumors, other platinum-resistant tumors downregulate PHGDH and rewire their metabolism toward NAD^+^-dependent pathways that sustain PARP activity and DNA repair. Rather than treating these observations as contradictory, we integrate them as two facets of the metabolic plasticity that OC cells exploit to survive therapeutic stress. We discuss how this context-dependent behavior conditions the rational use of PHGDH inhibitors, alone or in combination with platinum agents and PARP inhibitors, and the patient-stratification strategies that its clinical translation will require.

## 2. Ovarian Cancer

OC is a heterogeneous group of malignant gynecologic neoplasms affecting the ovary, fallopian tubes, and peritoneum [1,2,3]. OC predominantly presents as an epithelial carcinoma (95%), whereas a smaller proportion arises from germ cells (3%) or sex cord–stromal cells (2%) [2,28]. Histologically, ovarian carcinomas are classified into five subtypes: high-grade serous (HGSOC), low-grade serous (LGSOC), mucinous (MOC), clear cell (CCOC), and endometrioid (EOC) [3], each exhibiting a distinct molecular profile characterized by recurrent mutations in *TP53*, *BRCA1/2*, *KRAS*, *BRAF*, *PIK3CA*, *PTEN*, and *ARID1A* genes [29].

Several risk factors associated with the development of OC have been described; among the modifiable factors are nulliparity, hormone replacement therapy, smoking, and high-fat diets [30]. In contrast, non-modifiable factors include advanced age, late menopause, a family history of early-onset breast or ovarian cancer, and mutations in the *BRCA1/2* genes that increase the risk of developing OC by 41–58% and 15–16.5%, respectively [30,31].

OC is associated with poor survival when diagnosed at advanced stages, with 5-year survival rates of 26% and 16% for stages III and IV [4]. Worldwide incidence and mortality rates have been estimated at 6.6 and 3.9 cases per 100,000 population, respectively [32]. Late diagnosis is mainly due to nonspecific clinical manifestations [33]. In addition, the complex histopathological and molecular heterogeneity of OC contributes to suboptimal therapeutic strategies and poor prognosis [29].

Given these clinical challenges, accurate disease staging is essential. The International Federation of Gynecology and Obstetrics (FIGO) proposes a staging system for OC comprising 4 stages based on the tumor’s anatomical extent. Staging is determined surgically by removing gynecologic organs and potentially metastatic tissues for histological analysis [32,34,35]. OC staging is crucial, as it determines not only prognosis but also the most appropriate treatment regimen [36].

## 3. Current Therapeutic Approaches for Ovarian Cancer

The standard therapeutic management of OC is based on a multimodal strategy that combines cytoreductive surgery with systemic chemotherapy. Optimal debulking surgery, defined as the complete resection of macroscopic disease, remains a critical determinant of patient outcome and is consistently associated with improved progression-free and overall survival [5]. In patients with advanced-stage disease who are not suitable candidates for primary surgery, neoadjuvant chemotherapy (NACT) followed by interval debulking surgery is commonly employed, offering outcomes comparable to primary surgery with reduced surgical morbidity [37].

First-line systemic treatment predominantly consists of platinum-taxane-based chemotherapy, with carboplatin and paclitaxel representing the current standard of care. This regimen achieves high initial response rates; however, most patients ultimately relapse, highlighting the intrinsic and acquired limitations of this approach [5]. Based on the platinum-free interval, recurrent OC is clinically categorized as platinum-sensitive, partially platinum-sensitive, or platinum-resistant. This classification directly influences therapeutic decision-making and prognosis [6].

In the recurrent setting, platinum-sensitive disease is usually managed with platinum rechallenge. In contrast, platinum-resistant OC is treated with non-platinum agents, such as pegylated liposomal doxorubicin, gemcitabine, topotecan, or weekly paclitaxel. Despite this, response rates in platinum-resistant disease remain low, with a negligible effect on overall survival [5].

The therapeutic landscape of OC has expanded substantially with the introduction of targeted therapies. PARP inhibitors, including olaparib, niraparib, and rucaparib, have revolutionized treatment, particularly for patients harboring *BRCA1/2* mutations or homologous recombination deficiency (HRD) [38]. These agents exploit synthetic lethality by inhibiting DNA repair mechanisms, leading to sustained clinical benefit as maintenance therapy following platinum-based chemotherapy [39]. Importantly, PARP inhibitors have also demonstrated efficacy in patients without *BRCA* mutations, although predictive biomarkers beyond HRD remain under investigation [40].

Anti-angiogenic therapy has also been incorporated into the treatment of ovarian cancer. Bevacizumab, a monoclonal antibody targeting vascular endothelial growth factor (VEGF), has shown improvements in progression-free survival when administered in combination with chemotherapy and as maintenance therapy, particularly in patients with advanced-stage or high-risk disease [41]. However, the development of resistance and modest effects on overall survival remain essential limitations of this approach.

**Figure 1:**
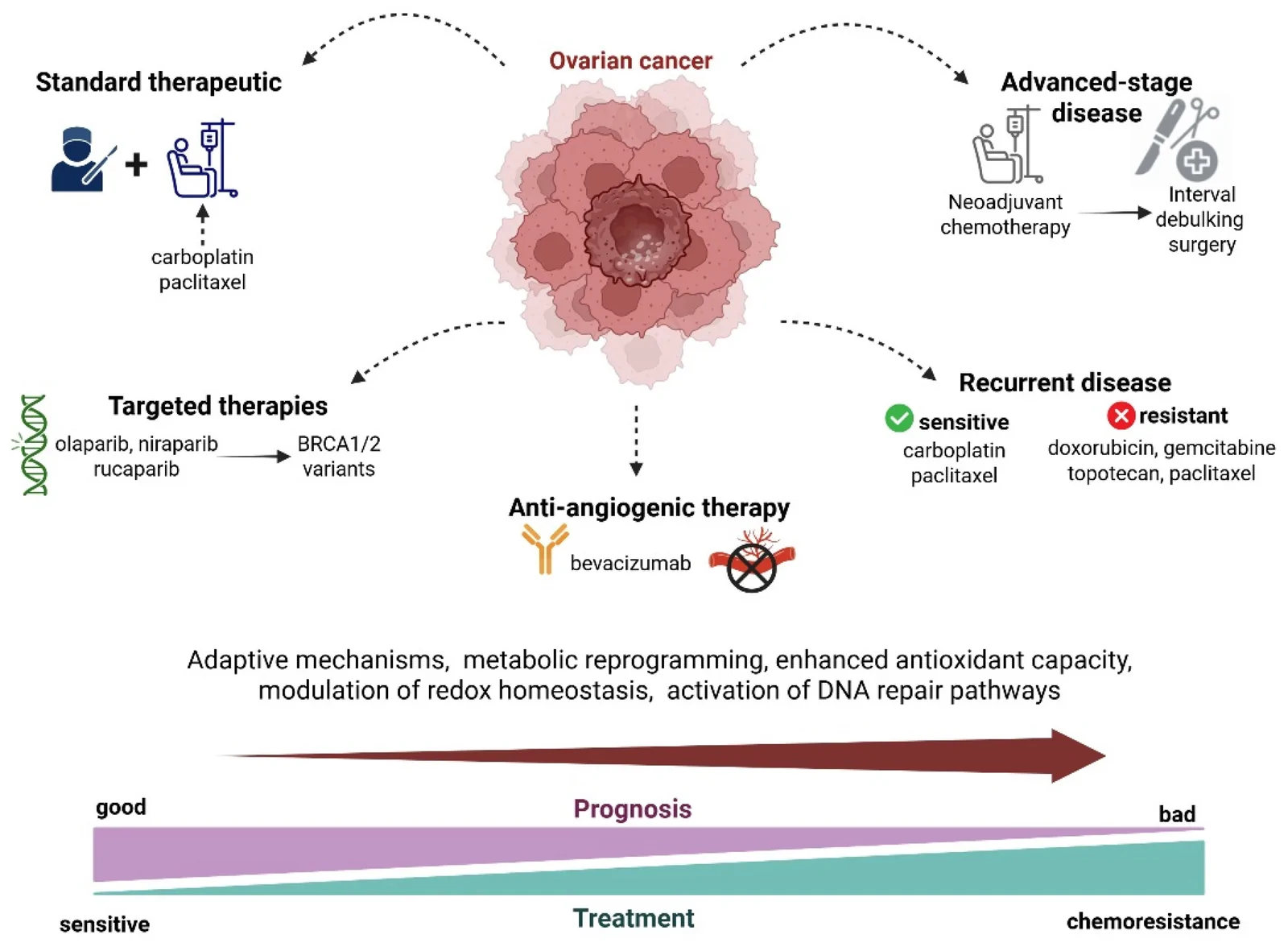
Current therapeutic approaches for OC. The main treatment strategies for OC consist of cytoreductive surgery combined with systemic chemotherapy based on platinum and taxanes. In advanced-stage disease, NACT is usually administered, followed by cytoreductive surgery. Patients who are resistant to platinum are treated with drugs such as doxorubicin, gemcitabine, topotecan and paclitaxel. Targeted therapies, such as PARP inhibitors, are available for carriers of *BRCA1/2* variants. Anti-angiogenic therapy has been incorporated as a strategy for treating advanced-stage OC in combination with chemotherapy. Due to the molecular mechanisms underlying disease progression or chemoresistance, it is necessary to implement new strategies that contribute to therapeutic efficacy, which in turn leads to a better prognosis. Created in BioRender. Apolonio, D. (2026) https://BioRender.com/9gou7eg (accessed on 22 October 2026).

Despite these advances, a substantial proportion of patients ultimately develop resistance to conventional therapy through adaptive mechanisms that compromise therapeutic efficacy [6,10], underscoring the need for novel strategies that target the underlying biology of resistant tumor cells (Figure 1), a phenomenon whose mechanisms are examined in detail below.

## 4. Mechanism of Chemoresistance in Ovarian Cancer

Chemoresistance is the primary barrier to long-term disease control in OC. Carboplatin and paclitaxel therapy, administered as adjuvant treatment in approximately 80% of patients, achieves high initial response rates, but is followed by disease recurrence in nearly 70% of women, closely linked to the development of chemoresistance [42].

Chemoresistance in OC is a multifactorial phenomenon involving several mechanisms, including evasion of apoptosis, epithelial-mesenchymal transition (EMT), upregulation of membrane drug efflux transporters, and epigenetic dysregulation [43,44,45]. Components of the tumor microenvironment, including cancer stem cells (CSCs), cancer-associated fibroblasts (CAFs), and tumor-associated macrophages (TAMs), further contribute to chemoresistance through intercellular communication [44,46].

Several of these mechanisms converge on the regulation of transcription factors such as NF-κB, PPARγ, p53, HIF-1α and NRF2, which govern the expression of genes involved in inflammation, apoptosis, cell proliferation and differentiation [47]. Notably, two of these factors act directly through metabolic reprogramming; NRF2 drives chemoresistance, proliferation and disease progression by regulating the antioxidant response [48], while HIF-1α promotes glycolytic flux and lactate production, thereby lowering reactive oxygen species (ROS) levels and preventing apoptotic cell death [42]. These observations position metabolic reprogramming not merely as a consequence of malignant transformation but as an active driver of therapeutic failure in OC, warranting closer examination.

## 5. Metabolic Reprogramming in Ovarian Cancer

Cancer is defined as a complex disease characterized by the uncontrolled growth of abnormal cells that share 14 acquired and facilitative functional capabilities collectively known as the hallmarks of cancer [49]. Among these capabilities, the reprogramming of cellular energy metabolism has emerged as a central feature of tumor biology, resulting from constant cell division that leads to the duplication of genetic material and the synthesis of new proteins, lipids, and other products [11]. Due to their high proliferation rate, tumor cells modify their metabolic pathways to meet the energy, biosynthetic, and redox balance needs arising from their malignant transformation [10]. Glycolysis, the tricarboxylic acid (TCA) cycle, fatty acid oxidation, and amino acid metabolism are energy pathways commonly altered in OC that promote cell survival and chemoresistance [50,51]. Changes in these pathways respond not only to the need to generate precursors for the biosynthesis of biomolecules but also to the requirement to maintain redox homeostasis by generating reducing power via NADPH and regulating ROS [52].

In HGSOC, enzymes of the TCA cycle are overexpressed; isocitrate dehydrogenase 1 (IDH1) promotes cell proliferation by reducing histone methylation in key cell cycle genes and is also associated with poorer survival, while citrate synthase (CS) provides the citrate necessary for de novo lipid synthesis via ATP-citrate lyase (ACLY) [51]. Meanwhile, alterations in lipid metabolism are characterized by increased lipogenesis and fatty acid oxidation, which contribute to the flexibility and fluidity of the cell membrane, as well as facilitating the cellular trafficking of components such as proteins and fatty acids [53]. In OC, lipogenesis is promoted by increased activity of enzymes such as fatty acid synthase (FASN), stearoyl-CoA desaturase 1 (SCD1), and Acetyl-CoA carboxylase (ACC), which is associated with a poor prognosis; furthermore, it has been reported that fatty acid oxidation increases during peritoneal dissemination, protecting OC cells from cell death by anoikis [51].

In OC tissues, particularly in the histological subtypes HGSOC and CCOC, overexpression of glycolytic enzymes such as pyruvate kinase M2 (PKM2), hexokinase 2 (HK2), and lactate dehydrogenase A (LDHA), as well as the regulators of glycolytic metabolism HIF-1α and STAT3, has been identified [50]. Additionally, glycolytic enzymes such as phosphoglycerate mutase 1 (PGAM1) have been associated with paclitaxel resistance by promoting pyruvic acid synthesis [54]. Aurora-A kinase promotes cisplatin resistance by dysregulating glycolytic enzymes such as LDHA and HK2 via SOX8/FOXK1 [55]. Additionally, aldolase A (ALDOA) and HK2 also promote cisplatin resistance via VEGFR2/STAT2 [56].

Furthermore, changes have been described in the metabolism of amino acids, particularly glutamine, which helps restore the antioxidant defense system, crucial for counteracting oxidative stress caused by the rapid growth and high metabolic activity of cancer cells [57]. In OC, glutaminase (GLS), an enzyme that uses glutamine as its substrate, is overexpressed and associated with poor prognosis [51]. Moreover, amino acid metabolism fuels multiple pathways involved in the maintenance of nonessential amino acids, protein synthesis, glucose and lipid metabolism, as well as nucleic acid biosynthesis [58]. Dysregulation of the metabolism of amino acids such as histidine, tryptophan, arginine, proline, alanine, glutamine, aspartate, glycine, and serine has been observed in cancer [59]. In particular, serine promotes tumor progression and growth [59] and can be synthesized de novo from glucose through a series of enzymatic reactions mediated by PHGDH, phosphoserine aminotransferase 1 (PSAT1), and phosphoserine phosphatase (PSPH) [60].

Notably, PHGDH has become especially significant, as its overexpression in OC is associated with poorer overall survival in patients and promotes spheroid formation, invasion, and chemoresistance to cisplatin in vitro models [14]. Consequently, there is growing interest in developing novel therapeutic strategies that complement existing treatments by targeting non-genomic vulnerabilities, including tumor metabolism. Enzymes involved in central carbon metabolism, nucleotide biosynthesis, and redox regulation have emerged as promising targets for combination therapies with platinum-based chemotherapy or PARP inhibitors [61,62]. Integrating metabolic-targeted approaches into current therapeutic regimens may therefore provide new opportunities to overcome chemoresistance and improve clinical outcomes in OC.

## 6. PHGDH: Structure, Function, Regulation, and Role in Cellular Metabolism

Glycolytic intermediates can be diverted from the main pathway to fuel alternative biosynthetic routes; specifically, 3-phosphoglycerate (3PG) is diverted to fuel the SSP to synthesize the nonessential amino acid serine [63]. PHGDH catalyzes the first step in the de novo SSP, which is crucial for the synthesis of a variety of biomolecules, including serine, glycine, and purine and pyrimidine nucleotides [64]. L-serine is a crucial amino acid as the precursor to D-serine. Still, it is also a precursor for the production of such metabolites as cysteine, phosphatidyl-L-serine, sphingolipids, purines, porphyrins, glyoxalate, and glycine. L-serine can also be produced from glycine by the action of serine hydroxymethyltransferase [65,66].

PHGDH is the first of three sequential enzymes in the SSP, followed by PSAT1 and PSPH [67]. The human *PHGDH* gene is located at 1p12 and encodes a 56.6 kDa protein with a 533-amino-acid open reading frame (ORF) [68]. PHGDH is present in a broad range of organisms, from bacteria to mammals, and exists in at least three distinct structural forms (types I, II, and III) [60,69]. Type I is dominant in humans, rodents, fungi, plants, and bacteria, while types II and III are primarily found in bacteria [70]. Human PHGDH possesses the type I structural form, consisting of two substrate-binding domains flanking a nucleotide-binding domain (NBD), together with two regulatory domains, the allosteric substrate-binding (ASB) and the aspartate kinase-chorismate mutase-tyrosine A prephenate dehydrogenase (ACT) domain [70].

Enzymes are remarkably efficient and specific, but can exhibit enzyme promiscuity, catalyzing a fortuitous side reaction in addition to their primary responses under certain circumstances and producing structurally similar substrates. There are identical structural features of the PHGDH substrate, 3-PHP, to other central metabolites, including pyruvate (lacks the phosphate of 3-PHP) and oxaloacetate [71]. These structural similarities among substrates and their related enzymes may interact with or be influenced by metabolic intermediates in glycolysis and the TCA cycle, potentially linking serine biosynthesis to broader metabolic networks. These enzymes have the potential to induce aberrant activities due to overexpression or abnormal stimulation. This phenomenon is exemplified by PHGDH, which exhibits additional enzymatic activity by converting the NADH-dependent reduction of α-ketoglutarate (α-KG) to the oncometabolite D-2-hydroxyglutarate (D-2HG) [60]. D-2HG is considered an oncometabolite, an aberrant metabolite found in tumor cells, exerting a pivotal influence on cancer [71]. Elevated levels of D-2HG inhibit α-KG-dependent dioxygenases (α-KGDDs), contributing to tumorigenesis by disrupting epigenetic regulation, genome integrity, hypoxia-inducible factor (HIF) signaling, and cellular metabolism [72].

It has been reported that active serine synthesis is required for the synthesis of proteins, lipids, and nucleotides, as well as for maintaining folate metabolism and redox homeostasis, both of which are necessary for cancer cells [60]. All these processes allow PHGDH to not only influence the growth and progression of cancer but also play an essential role in metastasis and drug resistance: pathway-derived chemoresistance mechanisms consist of the generation of products from 1C metabolism that contribute to nucleotide synthesis, redox balance via GSH synthesis and epigenetic regulation through methylation reactions [70]. Indeed, nucleotide synthesis and GSH antioxidant activity have been shown to mitigate the pharmacological action of platinum agents [73]. Specifically, GSH can act as a cofactor for multidrug resistance-associated protein 2 (MRP2), facilitating the efflux of chemotherapeutic drugs from the cell [74], and, through the action of glutathione-S-transferase-T1 (GSTT1), can conjugate with various xenobiotics, reducing their bioavailability and promoting platinum and taxol resistance in OC [75].

PHGDH undergoes several post-translational modifications, including phosphorylation, acetylation, methylation, palmitoylation, sumoylation, and lactylation [76]. Protein phosphorylation has been extensively studied, and in cancer, it has been linked to the dysregulation of several cellular processes associated with tumor progression. It has been observed in pancreatic cancer that glucose restriction induces the phosphorylation of PHGDH by p38 at Ser371 and by AMPK at Ser55, promoting the translocation of PHGDH from the cytoplasm into the nucleus, and, once in the nucleus, it sustains tumor growth under nutrient stress [77]. In drug-induced liver injury, PHGDH is phosphorylated at Ser371 by MAPK13 in response to oxidative stress, triggering its degradation via the chaperone-mediated autophagy pathway, attenuating GSH production, and leading to intracellular ROS accumulation [78].

Although most cancer types are associated with high PHGDH expression, low expression does not necessarily imply reduced oncogenic potential, as some tumors with low PHGDH levels remain highly aggressive [79]. The production of oncometabolites serves as a mediator of molecules such as GSH. Also, it mediates the production of other functions derived from post-translational modifications, which remain to be explored in various cancers.

## 7. Context-Dependent Role of PHGDH in Cancer

A detour of glycolysis towards serine synthesis drives anabolic pathways in central carbon metabolism by redistributing 1C units derived from serine and glycine [12], enabling the synthesis of metabolites to maintain epigenetic balance and cellular redox state [13,70]. Accordingly, PHGDH overexpression increases intracellular serine availability, driving cell proliferation, biomass generation, cancer progression, and therapy resistance [73].

This oncogenic contribution has been documented across a broad range of malignancies. In gastric cancer, PHGDH sustains the mitochondrial folate cycle to generate NADPH and counteract oxidative stress, a process regulated by ATF4/CEBPB that maintains cell pluripotency and drives chemoresistance [80]. In pancreatic ductal adenocarcinoma, silencing of AKR7A3 triggers aberrant PHGDH activation and impairs autophagy, positioning the AKR7A3-PHGDH axis as a key driver of chemoresistance [81].

In breast cancer, PHGDH additionally catalyzes the NADH-dependent reduction of α-KG to the oncometabolite D-2HG, promoting tumor progression [71]. In triple-negative breast cancer, PRMT1-mediated PHGDH methylation redirects metabolic flux toward serine synthesis and de novo fatty acid synthesis via FASN and S-palmitoylation, likewise promoting a chemoresistant phenotype [17]. Consistent with this oncogenic role, PHGDH knockdown restores doxorubicin sensitivity in triple-negative breast cancer by depleting GSH and increasing ROS accumulation [82].

In colorectal cancer, elevated PHGDH expression correlates with advanced Tumor-Node-Metastasis (TNM) stage and larger tumor size [21] and potentiates Hedgehog (HH) signaling to confer 5-fluorouracil (5-FU) resistance, an effect reversed by disrupting the PHGDH-HH axis [15].

In glioma cells, PHGDH activation inhibits ROS accumulation and promotes self-renewal by modulating redox homeostasis and 1C metabolism [83]. In acute myeloid leukemia, RNAi-mediated PHGDH silencing induces apoptosis through Bax upregulation and Bcl-2 downregulation, improving prognosis [84].

Paradoxically, however, PHGDH downregulation is not uniformly beneficial: in several contexts, reduced PHGDH is itself associated with a more aggressive or resistant phenotype. In breast cancer, PHGDH underexpression enhances metastatic potential by activating the hexosamine-sialic acid pathway, thereby promoting aberrant glycosylation and cell invasion [57]. Similarly, in osteosarcoma, RFWD3-mediated ubiquitination and degradation of PHGDH preserves NAD^+^ levels and supports nucleotide biosynthesis, which ultimately enhances chemoresistance [85]. Thus, even when PHGDH itself is downregulated, tumor cells appear to engage compensatory metabolic pathways to survive therapeutic responses and the subsequent emergence of resistance [80].

Collectively, these findings demonstrate that PHGDH’s role in cancer is not fixed but highly context-dependent, shaped by tumor type, the level of regulation involved, and the specific metabolic and therapeutic pressures a tumor faces. Table 1 summarizes the cancers in which PHGDH overexpression or downregulation has been reported, along with their principal biological consequences.

## 8. The Involvement of PHGDH in Chemoresistance in Ovarian Cancer

Having established that PHGDH’s contribution to malignancy varies widely across tumor types, we now turn to its specific role in OC, where metabolic reprogramming has been recognized as a hallmark of chemoresistance. PHGDH overexpression has been documented in different histological subtypes of OC, including HGSOC, MOC, CCOC, and EOC, with increased expression observed in platinum-resistant cells and tissues compared to their sensitive counterparts [14]. This increase has been associated with a significant reduction in survival in patients with OC [14,25].

PHGDH-regulated chemoresistance mechanisms include the generation of products from 1C metabolism that contribute to nucleotide synthesis, redox balance, and epigenetic regulation [70]. This antioxidant contribution is functionally significant. PHGDH overexpression increases GSH and NADPH levels, conferring resistance to chemotherapy-induced oxidative stress [14]. Consistent with this, induced PHGDH overexpression in SKOV3 and A2780 cells confers resistance to cisplatin, enhances invasion, and increases spheroid formation [14].

The molecular mechanisms underlying PHGDH’s role in OC chemoresistance remain poorly explored; however, its overexpression in platinum-resistant cells has been reported to result from positive regulation of translation by the DEAD-box helicase 3 X-linked (DDX3X), a *PHGDH* mRNA-binding protein, and the lncRNA RMRP [14]. Beyond this transcriptional and translational control, PHGDH activity is further modulated by post-translational modifications. Monoubiquitination at Lys146 promotes enzyme tetramerization and enhances its catalytic activity, increasing S-adenosylmethionine (SAM) levels and promoting metastasis [57,96]. Similarly, methylation at Arg236 enhances its catalytic activity and contributes to maintaining redox balance [97], suggesting that this layer of regulation may further shape PHGDH’s role in OC chemoresistance.

Downstream, PHGDH inhibition decreases levels of β-catenin, c-MYC, cyclin D1, PCNA, Bcl-2, N-cadherin, vimentin, and Snail [87], implicating PHGDH in activating pathways that promote EMT and cancer metastasis [75,88,98]. PHGDH regulation may also be integrated into adaptive metabolic programs mediated by redox-responsive factors. It has been shown that NRF2 overexpression in SKOV3 cells increases oxygen consumption rate (OCR) and promotes platinum resistance by inducing antioxidant enzymes such as glutamate-cysteine ligase catalytic subunit 1 (GCLC), NAD(P)H:quinone oxidoreductase 1 (NQO1), glucose-6-phosphate dehydrogenase (G6PDH), heme oxygenase 1 (HMOX1), and superoxide dismutase 2 (SOD2) [99]. Consistently, NRF2 has been shown to regulate, via ATF4, the expression of key enzymes in the SSP, including PHGDH, PSAT1, and serine hydroxymethyltransferase 2 (SHMT2) [100], suggesting that PHGDH may be part of an NRF2/ATF4-dependent metabolic program that supports tumor adaptation to chemotherapy-induced oxidative stress. Beyond tumor cells themselves, PHGDH has also been found to be overexpressed in CAFs cocultured with CAOV3 cells, supporting a role in tumor microenvironment-mediated OC progression [101].

**Figure 2:**
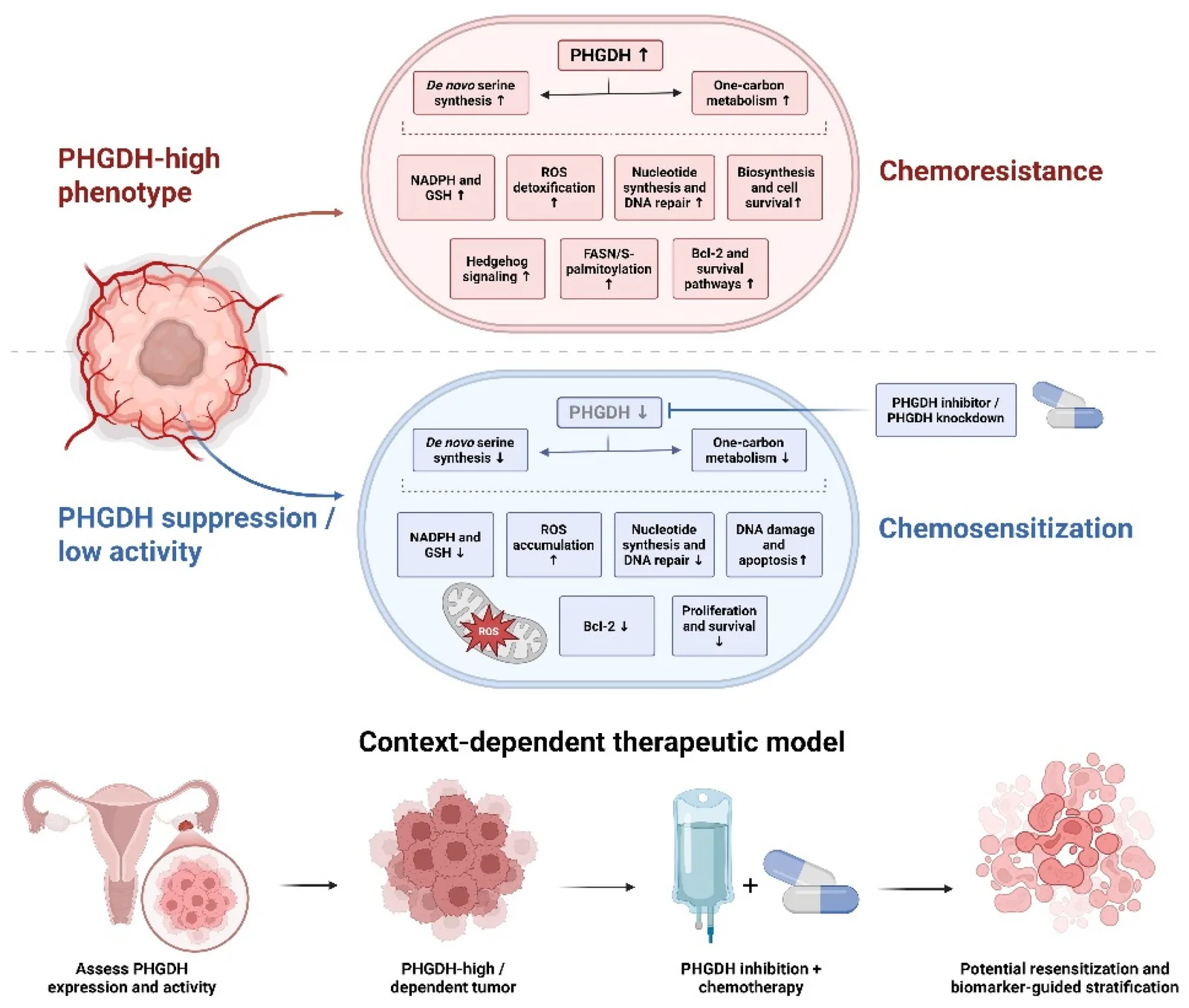
Dual and context-dependent contribution of PHGDH to the chemotherapy response. High PHGDH activity promotes de novo serine synthesis and 1C metabolism, supporting antioxidant defenses, nucleotide synthesis and DNA repair, lipid biosynthesis, and cell-survival pathways that contribute to chemoresistance. In PHGDH-dependent tumors, genetic knockdown or pharmacological inhibition of PHGDH reduces these metabolic and survival mechanisms, leading to ROS accumulation, DNA damage, apoptosis, and increased sensitivity to platinum and other anticancer therapies. Created in BioRender. Apolonio, D. (2026) https://BioRender.com/76fn88t (accessed on 22 October 2026).

Consistent with an oncogenic role, experimentally inhibiting PHGDH reverses several of these phenotypes. Genetic and pharmacological inhibition partially restores cisplatin sensitivity, decreasing cell viability and inducing apoptosis in chemoresistant cell lines [27]. In preclinical studies, the selective inhibitor CBR-5884 reduced cell proliferation by decreasing endogenous serine synthesis, inducing cell cycle arrest and apoptosis, and increased oxidative stress while augmenting Wnt/β-catenin pathway activity and enhancing the effect of the PARP inhibitor olaparib, and carboplatin sensitivity was associated with downregulation of stemness-related genes, including CD44, ALDH1A1, NANOG, SOX2, EPCAM, and POU5F1, in SKOV3 and ID8 cells [23]. CBR-5884 also reduces cancer cell viability in metabolic contexts that depend heavily on the serine pathway, even though it has not yet been evaluated in clinical trials [26]. Consistently, increased ROS generation and decreased NADPH and GSH levels were also observed in A2780, OVCAR3, and ES2 cells [24], and PHGDH inhibition before carboplatin exposure has been reported to enhance drug sensitivity in OC cells, indicating a critical role for PHGDH in intrinsic chemoresistance [6] (Figure 2). Accordingly, SKOV3 cells have been reported to exhibit lower sensitivity to platinum-based compounds compared to other OC cell lines, supporting its use as a model of intrinsic resistance and suggesting a potential association between this phenotype and PHGDH-mediated metabolic reprogramming [102,103].

Notably, not all evidence points toward a uniformly oncogenic role for PHGDH in OC. Recent data have reported low levels of PHGDH after relapse in some patients with resistance to platinum treatment; this reduction is accompanied by NAD^+^ production and sustained PARP activation, suggesting an alternative metabolic pathway that favors DNA repair and cancer cell survival [6].

### PHGDH and the Response to Radiotherapy

Both arms of this dual behavior converge on redox homeostasis and DNA repair, the same two processes that determine the cellular response to ionizing radiation. This raises the question of whether PHGDH also conditions radiosensitivity. Radiation therapy is a critical nonsurgical treatment for numerous cancer types because it induces radiolysis of water molecules, generating large amounts of free radicals and ROS that damage DNA, lipids, and proteins, producing metabolic and functional changes and ultimately apoptosis [104,105,106,107]. In OC, whole abdominal radiotherapy was initially employed for metastatic disease, but adverse effects and poor treatment compliance led to its withdrawal from conventional management; it nevertheless remains an option in refractory disease [108,109,110].

Radiation resistance, whether intrinsic or acquired, occurs in a substantial proportion of tumors and is a leading cause of local recurrence and treatment failure [111]. The underlying mechanisms include efficient DNA repair, tumor hypoxia, the persistence of CSCs, and evasion of apoptosis [112]. Metabolic plasticity is central to several of these processes, enabling tumor cells to meet their bioenergetic, biosynthetic and redox demands under hypoxia and nutrient restriction [10,113]. Targeting the metabolic pathways that sustain this homeostasis, particularly those governing oxidative stress buffering, has therefore been proposed as a strategy to enhance radiosensitivity.

The role of PHGDH in radiotherapy response remains poorly characterized, and the available evidence is both limited to non-ovarian models and apparently discordant. In lung cancer, PHGDH expression correlates positively with hypoxia gene expression signatures in patient datasets and is induced under severe hypoxia (<0.1% O_2_) through combined HIF-1 and PERK signaling; yet CRISPR-Cas9 deletion of PHGDH did not enhance radiosensitivity under either normoxia or hypoxia when extracellular serine and glycine were available at physiological concentrations. Radiosensitization was observed only under complete serine and glycine deprivation. Unexpectedly, PHGDH loss conferred greater tolerance to hypoxic stress, accompanied by increased glycolytic flux, elevated lactate production and accelerated HIF-1α stabilization [114]. In colorectal cancer, by contrast, pharmacological PHGDH inhibition radiosensitized cells under hypoxic conditions without altering their intrinsic radiosensitivity, an effect attributed to increased intracellular ROS and mitochondrial dysfunction; preliminary additive effects with radiotherapy were also observed in xenografts [115]. In glioblastoma, PHGDH is transcriptionally driven by c-MYC and supports redox homeostasis, 1C metabolism and the DNA damage response; accordingly, both genetic ablation and pharmacological inhibition suppressed tumor growth and sensitized orthotopic and patient-derived xenografts to radiotherapy [83]. These apparently conflicting observations are best reconciled by the availability of extracellular serine; where uptake can compensate for the loss of de novo synthesis, PHGDH inhibition alone is insufficient to radiosensitize, whereas tumors with a genuine dependency on the SSP, such as c-MYC-driven glioblastoma stem cells, do respond. Microenvironmental context is thus decisive, and PHGDH may function as a marker of a hypoxic, radioresistant niche as much as an autonomous mediator of radioresistance.

To date, no study has examined the effect of ionizing radiation on PHGDH expression or activity in OC. This represents a significant gap, given that PHGDH is upregulated in platinum-resistant OC cells and tissues [14], and that platinum resistance and radioresistance converge on shared DNA repair and redox mechanisms. Selective PHGDH inhibitors such as CBR-5884, which reduce stemness and enhance chemosensitivity in epithelial OC cells with high PHGDH expression [23], provide a tractable tool with which to address it. Further work is required to establish whether the de novo SSP is activated following irradiation in this tumor type and identify predictive biomarkers of serine pathway dependency that can distinguish patients who would benefit from combining serine/glycine-targeted agents with radiotherapy. Until then, PHGDH remains an unexplored target for radiosensitization in OC.

## 9. Targeting PHGDH: A Therapeutic Avenue in Ovarian Cancer

The paradoxical behavior of PHGDH in platinum-resistant OC underscores the therapeutic complexity of targeting this enzyme [6]. In a study analyzing 306 tumor samples from HGSOC patients who underwent debulking surgery and chemotherapy, high PHGDH expression was associated with lower overall survival and higher FIGO stage; furthermore, NACT was administered more frequently in patients with high PHGDH expression [25]. It has been hypothesized that tumors with high PHGDH expression are initially sensitive to chemotherapy but that surviving cells may undergo a metabolic shift toward adaptation and resistance, leading to disease relapse [6,25]. This oncogenic role is further supported functionally. Using a cisplatin-resistant model of SKOV3 and A2780 cells, PHGDH knockdown decreased cell survival, invasion, and spheroid formation; mechanistically, lncRNA RMRP upregulation in cisplatin-resistant cell lines leads to enrichment of DDX3X at *PHGDH* mRNA, which, in turn, enhances translation [14].

Distinct strategies have been developed to target the SSP by disrupting PHGDH, with a particular focus on allosteric and orthosteric inhibitors and their effects on catalytic activity [116]. However, the lack of a fully experimentally resolved PHGDH structure limits studies aimed at identifying new allosteric sites [117,118]. Many efforts have therefore relied on in silico approaches to calculate docking energies and protein structure and stability in the search for new inhibitory molecules [119]. Several compounds with potential inhibitory effects on PHGDH have been developed in recent years; however, none have yet reached clinical trials, underscoring the need for novel inhibitor discovery approaches [120]. One such preclinical example is withangulatin A (WA), a recently identified covalent inhibitor that selectively binds to Cys295, blocking the substrate-binding domain and causing allosteric inactivation; this reduced GSH synthesis, elevated ROS, and inhibited tumor proliferation [121].

The principal compound to be studied in OC is CBR-5884, an allosteric covalent inhibitor of PHGDH. As noted above, in A2780, OVCAR3, and ES2 cells, CBR-5884 inhibits proliferation, migration, and invasion by activating the ROS/Wnt/β-catenin pathway, and its combination with the PARP inhibitor olaparib enhances this therapeutic effect [24]. Complementing these findings, using spheroids, tumor xenografts, and patient-derived organoids (PDO), PHGDH inhibition by CBR-5884 has been shown to suppress the SSP and partially repress cellular steroid hormone biosynthesis, nucleotide biosynthesis, and 1C metabolism, altering the metabolic profile of OC cells [23].

OC cells continuously adapt to evade conventional treatments, and elucidating the mechanism underlying this adaptability may allow resistant phenotypes to be reversed and patient outcomes improved. Beyond its role in the SSP, PHGDH also participates in gene expression regulation, a still poorly understood function that may further inform future targeting strategies [122].

## 10. Conclusions and Future Perspectives

OC remains a highly lethal gynecologic malignancy, mainly due to late diagnosis and the frequent emergence of resistance to platinum-based chemotherapy. Accumulating evidence demonstrates that metabolic reprogramming is a central adaptive mechanism that enables OC cells to survive therapeutic stress, sustain tumor progression, and promote recurrence. Among the metabolic pathways implicated in this process, de novo SSP has emerged as a critical node linking cellular metabolism, redox homeostasis, epigenetic regulation, and DNA repair mechanisms.

PHGDH, as the rate-limiting enzyme of the SSP, plays a multifaceted role in OC biology. Beyond its canonical metabolic function, PHGDH contributes to tumor aggressiveness, stemness, EMT, and resistance to platinum compounds by sustaining antioxidant capacity, NADPH production, GSH synthesis, and 1C metabolism. Importantly, evidence indicates that PHGDH activity supports OC cell survival under chemotherapy-induced oxidative stress and facilitates adaptation to hostile metabolic environments.

However, emerging data also highlight the dynamic and tumor-specific nature of PHGDH regulation. While PHGDH overexpression is associated with poor prognosis and platinum resistance in a substantial subset of ovarian tumors, other tumors exhibit PHGDH downregulation following chemotherapy, accompanied by metabolic rewiring toward NAD^+^-dependent pathways and enhanced PARP-mediated DNA repair. This dual behavior underscores the metabolic plasticity of OC cells and suggests that PHGDH functions not merely as a static biomarker but as a flexible metabolic regulator during disease progression and treatment response.

Targeting PHGDH, therefore, represents a promising yet complex therapeutic strategy. Preclinical studies using genetic silencing or pharmacological inhibitors such as CBR-5884 have demonstrated reduced proliferation, increased oxidative stress, enhanced chemosensitivity, and synergistic effects with platinum agents or PARP inhibitors. Nevertheless, the limited clinical translation of PHGDH inhibitors reflects challenges, including metabolic compensation, tumor heterogeneity, insufficient selectivity, and an incomplete understanding of PHGDH’s non-canonical functions. These findings emphasize the need to stratify patients based on PHGDH expression, metabolic dependencies, and redox or DNA repair profiles before therapeutic intervention. The contribution of PHGDH to chemoresistance in OC is context-dependent, and identifying the specific conditions, molecular subtypes, and disease stages in which PHGDH targeting would be clinically beneficial remains an open and necessary line of investigation.

Future research should focus on several key directions. First, a deeper characterization of PHGDH post-transcriptional and post-translational regulation in OC is required to understand how its activity is modulated during chemotherapy and relapse. Second, integrative multi-omics approaches combining transcriptomics, metabolomics, and proteomics will be essential for identifying metabolic vulnerabilities in PHGDH-high and PHGDH-low tumor states. Third, rational combination therapies targeting PHGDH alongside DNA damage response pathways, redox regulators, or lipid metabolism may overcome compensatory mechanisms and improve therapeutic efficacy. Finally, the development of next-generation PHGDH inhibitors, guided by structural biology, computational modeling, and functional validation, will be critical to advancing these strategies toward clinical application.

In conclusion, PHGDH stands at the intersection of metabolic adaptation, chemoresistance, and tumor progression in OC. A comprehensive understanding of its context-dependent roles will be pivotal to the design of personalized metabolic therapies to overcome platinum resistance and improve patient outcomes.

## Figures and Tables

**Table 1:** Role of PHGDH in tumor progression, metabolic reprogramming, and chemotherapy resistance across different cancer types.

Cancer Type	Experimental Model	Study Type	PHGDH Expression	Biological Effect	Molecular Mechanism	Reference
Bladder cancer	T24 and RT4 cell lines; subcutaneous xenografts in BALB/c nude mice.	In vitro/In vivo	↑	Tumor progression and inhibition of ferroptosis.	PHGDH interacts with PCBP2 and stabilizes *SLC7A11* mRNA, thereby promoting its expression.	[86]
Osteosarcoma	Saos-2 and U-2OS cell lines.	In vitro	↑	Increased viability, migration, and invasion.	PHGDH maintains 1C metabolism and nucleotide biosynthesis independently of exogenous serine.	[87]
Hepatocellular carcinoma	MHCC-97H and HepG2 cell lines; orthotopic MHCC-97H liver xenografts in nude mice.	In vitro/In vivo	↑	Tumor progression, invasion, proliferation, and sorafenib resistance.	ZEB1 activates PHGDH transcription, increasing serine synthesis pathway flux and reducing oxidative stress.	[88]
Pancreatic cancer	PANC-1 and MIAPaCa-2 cell lines.	In vitro	↑	Invasion, migration, and resistance to 5-FU and oxaliplatin.	AKR7A3 inhibition increases PHGDH expression, thereby suppressing autophagy and promoting tumor progression.	[81]
Gastric cancer	HS746T and NCI-N87 cell lines; patient-derived gastric cancer organoids; HS746T peritoneal dissemination xenografts in BALB/c nude mice.	In vitro/In vivo	↑	Resistance to GLS inhibition and glutamine deprivation.	ATF4 and CEBPB induce PHGDH expression, promoting 1C metabolism and NADPH production to counteract ROS.	[80]
Triple-negative breast cancer	MDA-MB-468 and paclitaxel-resistant MDA-MB-231 cell lines; subcutaneous MDA-MB-231 xenografts in NOG mice.	In vitro/In vivo	↑	Paclitaxel resistance.	PHGDH is stabilized through PRMT1-dependent methylation at Arg20 and Arg54, promoting de novo fatty acid synthesis and S-palmitoylation.	[17]
Glioblastoma	Patient-derived GSC19 and GSC29 glioma stem cells; orthotopic intracranial xenografts in nude mice; subcutaneous PDX models in NOD/SCID mice.	In vitro/In vivo	↑	Tumor progression, glioma stem cell self-renewal, and radioresistance.	c-MYC induces PHGDH expression, promoting redox homeostasis and DNA damage response.	[83]
Lung adenocarcinoma	A549 and PC9 cell lines; subcutaneous A549 xenografts in BALB/c nude mice.	In vitro/In vivo	↑	Tumor progression and ferroptosis resistance.	ZNF146 transcriptionally induces PHGDH expression, sustaining GSH and NADPH levels to suppress lipid peroxidation and ferroptosis.	[89]
Colorectal cancer	SW620, HCT116, and HCT15 cell lines; subcutaneous SW620 xenografts in BALB/c nude mice.	In vitro/In vivo	↑	Tumor progression, migration, and invasion.	NSUN2 increases *PHGDH* mRNA stability in an ALYREF-dependent manner.	[90]
Colorectal cancer	HT29 and HCT116 cells co-cultured with THP-1-derived macrophages; subcutaneous HT29 xenografts in BALB/c nude mice.	In vitro/In vivo	↑	Resistance to 5-FU and oxaliplatin; TAM polarization toward an M2 phenotype.	CXCL7 induces PHGDH expression in a STAT1-dependent manner and promotes SAM production.	[91]
Colorectal cancer	HCT8 and RKO cell lines; patient-derived colorectal cancer organoids; subcutaneous HCT8 xenografts in *nu/nu* mice.	In vitro/In vivo	↑	Resistance to 5-FU, invasion, and cancer stem-like phenotype.	PHGDH overexpression activates Hedgehog signaling and its target genes, including VEGFA, ALDH1, ABCB1, and N-cadherin.	[15]
Colorectal cancer	HCT116 and SW620 cell lines.	In vitro	↑	Partial ferroptosis suppression.	ARNTL2 transcriptionally induces PHGDH, which binds and stabilizes *SLC7A11* mRNA.	[92]
Gastric cancer	HGC27 and MGC803 cell lines; subcutaneous HGC27 xenografts in BALB/c nude mice; patient-derived xenografts in NOD/SCID mice; MNU-induced gastric tumors in *Wnk1* conditional knockout mice.	In vitro/In vivo	↑	Ferroptosis inhibition, tumor progression, and oxaliplatin resistance.	WNK1 phosphorylates PHGDH at Ser349 and Ser371, increasing its stability and activity.	[93]
Colorectal cancer	HCT116 spheroid cultures; subcutaneous HCT116 xenografts in athymic nude mice.	In vitro/In vivo	↓	Metabolic alteration with toxic metabolite accumulation.	PHGDH inhibition promotes production of 1-deoxysphingolipids.	[94]
Breast cancer	4T1, EMT6.5, and MDA-MB-231 cell lines; orthotopic 4T1 and EMT6.5 mammary fat pad tumors in BALB/c mice.	In vitro/In vivo	↓	Migration, invasion, and metastasis.	Low PHGDH disrupts its interaction with PFKP, activating the hexosamine-sialic acid pathway and increasing integrin sialylation.	[57]
Breast cancer	THP-1-derived macrophages conditioned with MDA-MB-231 medium and mouse bone marrow-derived macrophages; orthotopic EO771 tumors co-injected with macrophages in C57BL/6 mice.	In vitro/In vivo	↓	Macrophage polarization toward an M2 phenotype and immunosuppressive function.	Loss of nuclear PHGDH in macrophages upregulates GLUD1 and GLS2 transcription via STAT3.	[95]
Osteosarcoma	143B and U-2OS cell lines; subcutaneous 143B xenografts in BALB/c nude mice.	In vitro/In vivo	↓	Cisplatin chemoresistance.	RFWD3 mediates PHGDH degradation via ubiquitination at Lys137, thereby elevating NAD^+^ levels that drive de novo nucleotide biosynthesis.	[85]

↓ indicates protein subexpression, ↑ indicates protein overexpression.

## Data Availability

No new data were created or analyzed in this study. Data sharing is not applicable to this article.

## Abbreviations

PHGDH	D-3-phosphoglycerate dehydrogenase
3PG	3-phosphoglycerate
3-PHP	3-phosphohydroxypyruvate
OC	Ovarian cancer
FIGO	International Federation of Gynecology and Obstetrics
PARP	Poly (ADP-ribose) polymerase
1C	One-carbon
NADPH	Reduced nicotinamide adenine dinucleotide phosphate
GSH	Glutathione
SSP	Serine synthesis pathway
HGSOC	High-grade serous ovarian carcinoma
LGSOC	Low-grade serous ovarian carcinoma
MOC	Mucinous ovarian carcinoma
CCOC	Clear cell ovarian carcinoma
EOC	Endometrioid ovarian carcinoma
NACT	Neoadjuvant chemotherapy
HRD	Homologous recombination deficiency
VEGF	Vascular endothelial growth factor
EMT	Epithelial-mesenchymal transition
CSCs	Cancer stem cells
CAFs	Cancer-associated fibroblasts
TAMs	Tumor-associated macrophages
NF-κB	Nuclear factor kappa-light-chain-enhancer of activated B cells
PPARγ	Peroxisome proliferator-activated receptor gamma
HIF-1α	Hypoxia-inducible factor 1-alpha
NRF2	Nuclear factor erythroid 2–related factor 2
ROS	Reactive oxygen species
TCA	Tricarboxylic acid
IDH1	Isocitrate dehydrogenase 1
CS	Citrate synthase
ACLY	ATP-citrate lyase
FASN	Fatty acid synthase
SCD1	Stearoyl-CoA desaturase 1
ACC	Acetyl-CoA carboxylase
PKM2	Pyruvate kinase M2
HK2	Hexokinase 2
LDHA	Lactate dehydrogenase A
STAT3	Signal transducer and activator of transcription 3
PGAM1	Phosphoglycerate mutase 1
ALDOA	Aldolase A
VEGFR2	Vascular endothelial growth factor receptor 2
STAT2	Signal transducer and activator of transcription 2
GLS	Glutaminase
PSAT1	Phosphoserine aminotransferase 1
PSPH	Phosphoserine phosphatase
ORF	Open reading frame
NBD	Nucleotide-binding domain
ASB	Allosteric substrate-binding domain
ACT	Aspartate kinase-chorismate mutase-tyrosine A prephenate dehydrogenase domain
α-KG	α-ketoglutarate
D-2HG	D-2-hydroxyglutarate
α-KGDD	α-KG-dependent dioxygenase
HIFs	Hypoxia-inducible factors
MRP2	Multidrug resistance-associated protein 2
GSTT1	Glutathione-S-transferase-T1
TNM	Tumor-Node-Metastasis
HH	Hedgehog
5-FU	5-fluorouracil
DDX3X	DEAD-box helicase 3 X-linked
SAM	S-adenosylmethionine
OCR	Oxygen consumption rate
GCLC	Glutamate-cysteine ligase catalytic subunit 1
NQO1	NAD(P)H:quinone oxidoreductase 1
G6PDH	Glucose-6-phosphate dehydrogenase
HMOX1	Heme oxygenase 1
SOD2	Superoxide dismutase 2
SHMT2	Serine hydroxymethyltransferase 2
WA	Withangulatin A
PDOs	Patient-derived organoids
PCBP2	Poly(C)-binding protein 2
SLC7A11	Solute carrier family 7 member 11
ZEB1	Zinc finger E-box binding homeobox 1
VEGFA	Vascular endothelial growth factor A
ALDH1	Aldehyde dehydrogenase 1
ABCB1	ATP-binding cassette subfamily B member 1
WNK1	WNK lysine deficient protein kinase 1
PFKP	Phosphofructokinase, platelet type
GLUD1	Glutamate dehydrogenase 1
GLS2	Glutaminase 2
NSUN2	NOP2/Sun RNA methyltransferase 2
ALYREF	Aly/REF export factor
CXCL7	C-X-C motif chemokine ligand 7
STAT1	Signal transducer and activator of transcription 1
c-MYC	MYC proto-oncogene
ZNF146	Zinc finger protein 146
ARNTL2	Aryl hydrocarbon receptor nuclear translocator like 2
